# Strategies to Prevent Anthracycline-Related Congestive Heart Failure in Survivors of Childhood Cancer

**DOI:** 10.1155/2012/713294

**Published:** 2012-08-15

**Authors:** Saro H. Armenian, Sarah K. Gelehrter, Eric J. Chow

**Affiliations:** ^1^Department of Population Sciences, City of Hope Comprehensive Cancer Center, 1500 East Duarte Bovlevard, Duarte, CA 91010, USA; ^2^Division of Pediatric Cardiology, Department of Pediatrics and Communicable Diseases, C.S. Mott Children's Hospital, University of Michigan, Ann Arbor, MI 48109, USA; ^3^Clinical Research and Public Health Science Divisions, Fred Hutchinson Cancer Research Center, Seattle, WA 98109, USA; ^4^Department of Pediatrics, Seattle Children's Hospital, Seattle, WA 98105, USA

## Abstract

Cardiovascular complications are a leading cause of therapy-related morbidity and mortality in long-term survivors of childhood malignancy. In fact, childhood cancer survivors are at a 15-fold risk of developing CHF compared to age-matched controls. There is a strong dose-dependent association between anthracycline exposure and risk of CHF, and the incidence increases with longer followup. Outcome following diagnosis of CHF is generally poor, with overall survival less than 50% at 5 years. The growing number of childhood cancer survivors makes it imperative that strategies be developed to prevent symptomatic heart disease in this vulnerable population. We present here an overview of the current state of knowledge regarding primary, secondary, and tertiary prevention strategies for childhood cancer survivors at high risk for CHF, drawing on lessons learned from prevention studies in nononcology populations as well as from the more limited experience in cancer survivors.

## 1. Introduction

Anthracyclines (doxorubicin, daunomycin, idarubicin, epirubicin, and mitoxantrone) are widely used in the treatment of childhood cancer; the use of these agents has led to significant advances in the outcome of many childhood cancers [1]; current 5-year survival rates exceed 80% [2]. Clinically, one of the most widely recognized side-effects of anthracycline therapy is dose-dependent cardiotoxicity, which manifests along a continuum from asymptomatic cardiac dysfunction identified by abnormalities of cardiac function/structure detected on imaging studies, to clinically overt congestive heart failure (CHF) [1]. The incidence of CHF is less than 5% with cumulative anthracycline exposure of <300 mg/m²; approaches 15% at doses between 300 and 500 mg/m²; exceeds 30% for doses >600 mg/m² [3–7]. In addition, clear associations have been established between clinical variables and risk of therapy-related CHF; these variables include younger age (<5 years) at exposure, female gender, preexisting heart disease, and concomitant mediastinal irradiation [7, 8]. Further increasing lifetime risk for development of CHF, survivors of childhood cancer are at a higher risk of developing cardiovascular risk factors such as hypertension and diabetes compared with age-and sex-matched controls [9]. Outcome following diagnosis of CHF is generally poor, with overall survival of less than 50% at 5 years [10]. Current estimates indicate that nearly 60% of the 350,000 survivors of childhood cancer in the US will have been treated with anthracyclines [5, 11]—a vulnerable subpopulation at risk for symptomatic heart disease, and therefore representing a critical need for prevention strategies to decrease/reverse this morbidity.

**Figure 1:**
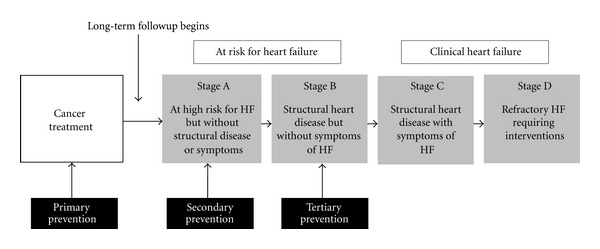
Heart failure (HF) prevention strategies, modified from the ACC/AHA guidelines.

The American College of Cardiology/American Heart Association (ACC/AHA) guidelines for the diagnosis and management of CHF describe it as a progressive disorder (Figure 1) [12]. Left ventricular (LV) dysfunction begins with some injury to, or stress on, the myocardium (stage A) and may be progressive even in the absence of a new identifiable insult to the heart. The eventual manifestation is a change in the geometry or structure of the left ventricle (stage B) which precedes clinically overt disease (stage C/D). According to the ACC/AHA guidelines for management of CHF, [12] patients either remain in their current stage or advance from one stage to the next, but do not revert back to an earlier stage. It is well recognized that there is a long latency between asymptomatic (stage A/B) and clinically evident (stage C/D) disease in childhood cancer survivors exposed to high-dose anthracyclines [8, 13]. Over time, anthracycline exposure leads to a decrease in LV wall thickness, increase in LV dimension, and subsequent increase in LV end-systolic wall stress (ESWS)-a critical component of myocardial remodeling and neurohormonal imbalance that precedes CHF [4].

The well-characterized natural history of cardiac dysfunction after anthracycline exposure in childhood provides clinicians with unique opportunities to explore paradigms for disease prevention. We present here an overview of the current knowledge regarding primary, secondary, and tertiary prevention strategies in patients at high risk for CHF (Table 1). We believe that a clear understanding of those at highest risk due to established risk factors as well lessons learned from non-oncology populations will set the stage for future studies that will comprehensively address risk reduction in a vulnerable population of survivors.

## 2. Primary Prevention

 The most effective approach to minimizing cardiotoxicity due to anthracyclines has been the reduction of lifetime cumulative dose for children with malignancies that have favorable outcomes such as Hodgkin lymphoma and acute lymphoblastic leukemia. However, for other malignancies such musculoskeletal tumors and acute myelogenous leukemia, high-dose anthracyclines remain the backbone of most contemporary treatments. As a result, the oncology community has continued to explore novel treatment approaches that preserve treatment efficacy while reducing cardiotoxicity, including (1) less cardiotoxic anthracycline derivatives; (2) alternative administration schedules; (3) concurrent administration of a cardioprotectant. The results of selected randomized clinical trials (RCTs) examining these strategies have been reviewed in several meta-analyses [14–17] and are also summarized here. Of note, the majority of published RCTs enrolled adults, mostly patients with breast cancer and other solid tumors, and few included pediatric patients.

 Doxorubicin and daunomycin analogs that have been designed to reduce cardiotoxicity while preserving antitumor effect include epirubicin and idarubicin, respectively [17]. Mitoxantrone is an anthraquinone (anthracenedione) derivative designed with a similar goal. The majority of available RCTs have compared doxorubicin versus epirubicin [16]. Epirubicin was associated with an approximate 60% decreased risk of clinical heart failure compared with doxorubicin in two separate meta-analyses (*P* value ranged from <0.01 to 0.07), with no reduction in antitumor efficacy [16, 17]. Two trials have compared doxorubicin or epirubicin with idarubicin among lymphoma patients and did not report differences in cardiotoxicity or tumor efficacy [18, 19]. Various RCTs have examined the relative effects of mitoxantrone compared with doxorubicin or epirubicin [16, 17]. Overall, these studies appear to suggest a 3-fold decreased risk of clinical cardiotoxicity associated with mitoxantrone, although analyses restricted to subclinical outcomes, such as asymptomatic fall in LV ejection fraction, did not show a significant effect.

Liposomal formulations of doxorubicin (and less commonly, daunomycin) have been developed based on the premise that they are less likely to extravasate from normal vasculature and be taken up by healthy tissue [20]. Studies conducted mostly in adult cancer patients appear to show a strong protective effect associated with liposomal doxorubicin compared with the native form, with an estimated 80% reduction in clinical cardiac outcomes and a 60–70% decrease in subclinical outcomes [14, 17]. Of note, there were no differences in reported tumor response. There is a paucity of information regarding potential cardioprotective effects of liposomal doxorubicin in children treated with anthracyclines.

An alternative strategy to reduce therapy-related cardiotoxicity has been to increase the anthracycline infusion time with the hypothesis that reducing peak plasma concentrations reduces cardiotoxicity while preserving overall exposure (area under the curve) and antitumor efficacy [21]. Early adult studies have shown decreased histologic injury on myocardial biopsies and reduced incidence of clinical heart failure with prolonged infusion lengths upwards of 96 hours compared with bolus administration without compromising antitumor effect [14]. In RCTs, longer infusions of at least 6 hours were associated with 3- or 4-fold reduction in clinical heart failure versus bolus dosing without compromising antitumor efficacy [14]. On the other hand, a pediatric RCT [22] in children treated for leukemia found no difference in cardiac outcomes between those randomized to receive bolus versus continuous infusion of anthracycline. Similar null results have been reported in uncontrolled pediatric trials [23].

Finally, a variety of possible cardioprotectants including amifostine, acetylcysteine, calcium channel blockers, carvedilol, coenzyme Q10, and L-carnitine also have been tested [15, 16]. Although some results were suggestive of cardioprotection, they did not achieve statistical significance and therefore require additional investigation. At present, none of these agents are considered standard of care. On the other hand, the evidence for dexrazoxane (DRZ) as a cardioprotectant is more mature. DRZ is an EDTA-like bisdioxopiperazine that decreases oxygen free radicals via intracellular iron chelation among other activities [21]. Overall, summary risk estimates based on RCT data suggest that DRZ is associated with a significantly decreased risk of both clinical (relative risk ~0.2) and subclinical (relative risk ~0.3) heart failure during and shortly after therapy [13, 15, 16, 24]. In available studies, overall acute severe or life-threatening toxicities did not appear to be worse among DRZ-exposed versus unexposed patients, and there were no differences in tumor response rates [15]. While data again are mostly based on adult breast cancer patients, at least 2 RCTs in children suggest that DRZ may be effective in ameliorating cardiotoxicity [24–26]. In both trials, there were no statistical differences in cancer recurrence rates or overall survival between the study arms [25, 26]. Concerns regarding a possible association between DRZ and an increased risk of second cancers in children with Hodgkin lymphoma have hindered its more widespread use among children [27]. However, several subsequent well-powered randomized clinical trials in children with acute leukemia have not demonstrated interference with antitumor efficacy of anthracyclines, nor evidence of increased risk of second malignancy in children exposed to DRZ [26, 28–30].

Currently DRZ is approved for use by the US Food and Drug Administration only in women with metastatic breast cancer who have received 300 mg/m^2^ of anthracyclines and who may benefit from further anthracycline-based therapy, a recommendation largely supported by the American Society of Clinical Oncology [31].

### 2.1. Future Directions

 Although the available research to date suggests that certain anthracycline derivatives or administration schedules may be associated with a reduced risk of cardiotoxicity, adoption of these strategies has been limited. Long-term efficacy data is lacking, and certain subgroups, particularly children who have the greatest potential number of life years following cancer therapy, remain understudied. One barrier to wider adoption is the higher financial cost of most derivative formulations, particularly liposomal forms, and strategies that require a prolonged infusion time [32, 33]. Nevertheless, given the health and economic costs of potentially preventable cardiotoxicity, cost-effectiveness analyses may provide important data for health policy makers.

 Finally, although the cumulative dose (or perhaps the peak plasma concentration) likely remains the single most important factor in influencing anthracycline-related cardiotoxicity, some patients develop clinical sequelae at even relatively low doses while others do not appear to be affected despite very high doses, suggesting the importance of host-specific factors. There is emerging data to suggest that genetic susceptibility could play a role in modifying individual response to therapeutic exposures [34, 35]. Using a biologically plausible candidate gene approach, investigators have begun to identify polymorphisms that could alter metabolic pathways of therapeutic agents associated with specific adverse events, including CHF [34, 35]. Many of these genomic variables, when fully established, could be important in facilitating the implementation of targeted primary prevention strategies such as individualized therapy in survivors at highest risk for CHF. 

## 3. Secondary Prevention

 While it is anticipated that advances in our understanding of the pathophysiology of anthracycline-associated CHF may one day pave the way for personalized delivery of cancer care, there will continue to be a growing number of long-term survivors who remain at risk for CHF due to past exposure to cardiotoxic therapies. Monitoring of anthracycline-related cardiotoxicity in these at-risk survivors has relied upon serial echocardiographic screening using resting left ventricular ejection fraction (LVEF) or shortening fraction (LVSF) [36]. These parameters are derived from measurements of ventricular volume and size that have intrinsic limitations; even with optimal echocardiographic image quality, measurement of LVEF and LVSF makes assumptions about ventricular geometry, the measurements are load-dependent, and data on the accuracy and reproducibility of these measures in pediatric patients are limited. Importantly, LVEF and LVSF have increasingly been recognized as inadequate for detecting subtle changes in myocardial function [37, 38]. Often, at the point when changes in LVEF are detected, functional deterioration proceeds rapidly and is often irreversible, emphasizing the importance of prevention strategies in high-risk survivors prior to onset of systolic dysfunction.

It is increasingly recognized that survivors of childhood cancer are at a higher risk of developing cardiovascular risk factors such as hypertension and diabetes compared with age- and sex-matched controls [9]. In nononcology populations, hypertension confers a two-fold risk for the occurrence of CHF and carries the highest population attributable risk for CHF [39]. In animal models, there is evidence that hypertension may accelerate left ventricular myocardial remodeling known to occur following anthracycline exposure [40]. The pathophysiology of heart failure in patients with diabetes is more complex, and can be due to silent myocardial infarction or as a result of metabolic derangement due to hyperglycemia [41]. A recent study [42] in adult survivors of hematologic malignancy found that the presence of hypertension among recipients of anthracycline was associated with a 35-fold increased risk of CHF, while the risk was nearly 27-fold for anthracycline recipients who developed diabetes, providing further evidence that hypertension and diabetes may be critical modifiers of anthracycline-related left ventricular myocardial injury. These findings set the stage for developing novel paradigms for secondary prevention that includes behavior modification after cancer treatment (adoption of a healthy lifestyle and aggressive management of cardiovascular risk factors), and targeted early interventions for survivors at highest risk for CHF (e.g., female, high-dose anthracycline exposure, chest radiation, younger age at treatment) [13]. 

 Clinicians caring for childhood cancer survivors have been hesitant to use secondary pharmacologic strategies in asymptomatic “at risk” populations, in large part because of the paucity of well-conducted RCTs that would provide the evidence to support such an intervention. The only study to date evaluating efficacy of secondary intervention to prevent CHF was a randomized trial of afterload reduction in asymptomatic survivors exposed to any dose of anthracyclines [43]. While the study failed to demonstrate clinically significant improvement in cardiac function, investigators suggested that those previously treated with high dose (≥300 mg/m²) of anthracyclines benefited most from the intervention. Due to small numbers in the high-dose arm and relatively short followup of two years, investigators were unable to make more definitive recommendations regarding prevention.

 While the experience in secondary pharmacologic prevention in childhood cancer survivors is limited, lessons learned from other high-risk populations may pave the way for new approaches to CHF risk reduction after cancer treatment. Duchenne muscular dystrophy (DMD) is a genetic condition that is characterized by progressive muscle weakness and eventual cardiac involvement. Much like anthracycline-related CHF, cardiac involvement begins as minor electrocardiographic abnormalities at a young age, evolves toward cardiomyopathy with dilation of cardiac chambers and subsequent decrease in LVEF. Most develop refractory dilated cardiomyopathy, which is responsible for nearly 50% of patient deaths. Treatment with ACE-inhibitors or *β*-blockers has been shown to improve cardiac function among patients with Stage C disease and delay onset of refractory (stage D) disease [44]. However, few patients have lasting improvement in outcomes and most develop progressive disease despite pharmacologic intervention. The only long-term secondary prevention trial randomized children with DMD (range 9.5–13 years old) with preserved EF and no clinical evidence of CHF to afterload reduction with the ACE inhibitor perindopril *versus* placebo [45]. Median LVEF at study entry was 65.0% ± 5.5%. In this double-blinded, multicenter trial, overall survival in the perindopril arm was significantly superior to placebo at 10 years (93% *versus* 65.5%, *P* = 0.01). All deaths were due to cardiopulmonary failure. This study was one of the first to demonstrate the critical importance of long-term intervention prior to onset of clinically evident disease in patients with preserved LVEF, but known to be at high risk for developing CHF, a strategy that may be worth pursuing in high risk childhood cancer survivors.

### 3.1. Future Directions

 One of the recognized challenges of secondary prevention strategies in this population is the paucity of robust surrogate endpoints for assessment of response to an intervention in the setting of preserved LVEF [46, 47]. Therefore, ongoing studies to establish these endpoints are critical to the development of effective secondary prevention. The best studied blood biomarkers of myocardial injury and remodeling include cardiac troponins (cTn) and natriuretic peptides (NP). While cTn's have successfully been used to monitor acute anthracycline-related cardiotoxicity, [24] little is known regarding their utility for diagnosis and monitoring of long-term chronic cardiac injury [48]. cTn levels have failed to identify mild heart dysfunction in patients followed long-term, [49, 50] arguing against their use as a biomarker of response to pharmacologic intervention. NPs serve as independent risk factors for adverse cardiovascular events, and are being increasingly advocated as objective markers to monitor and adjust anticongestive treatment [51, 52]. However, there are limitations to their widespread use in asymptomatic patients due to their low specificity and wide variability in the measured value, determined both by the specific peptide assay as well as by physiologic conditions [48]. A recent study [53] reported that patients with elevated cTn's and NP's shortly after anthracycline administration are at increased risk of myocardial remodeling 4 years after completion of therapy, suggesting that patients who experience acute myocardial injury, as measured by blood biomarkers, may be at an especially high risk for late-occurring cardiac dysfunction, setting the stage for closer monitoring and subsequent interventions.

 While blood biomarkers exist, most evaluations of chronic myocardial remodeling are based on well-characterized serial echocardiographic measurements such as LV end-systolic wall stress (ESWS), [4] myocardial performance index (MPI), [54] and thickness-dimension ratio [4]. However, the prognostic utility of these intermediate indices in patients treated with anthracyclines is unknown due, in part, to lack of long-term followup in many of these studies. It remains to be seen if novel approaches to screening such as magnetic resonance imaging, tissue doppler imaging, “speckle tracking,” or 3D echocardiography will be able to provide us with more accurate endpoints necessary to measure response following interventions in high-risk childhood cancer survivors with preserved LVEF [55, 56].

 Currently, heart-healthy lifestyles are encouraged for all childhood cancer survivors, and this is especially relevant for survivors treated with anthracyclines [57]. Management of traditional cardiovascular risk factors includes implementation of a regular exercise program, dietary recommendations, counseling regarding the importance of avoiding or ceasing tobacco use, as well as screening and treatment of hypertension, diabetes, and dyslipidemia. It remains to be seen if early pharmacologic intervention with low-dose ACE inhibitors and/or betablockers in patients with preserved LVEF but at high-risk CHF due to other indices of myocardial remodeling will prevent the risk of subsequent CHF.

## 4. Tertiary Prevention

 In the nononcology community, there is universal agreement about the importance of initiating an appropriate intervention for patients with stage B heart failure [58, 59]. This approach has been widely applied to individuals with prior acute myocardial infarction as well as for those with other causes of LV dilatation and hypokinesia [58, 59]. The Carvedilol and ACE-inhibitor Remodeling Mild Heart Failure Evaluation (CARMEN) [60] trial randomized participants with mild heart failure to: enalapril, carvedilol, or both. LV remodeling was assessed by serial LV end-systolic volume index (LVESVI) measurements for 18 months. Carvedilol significantly reduced LVESVI compared to baseline, whereas enalapril did not; there was even greater reduction with the combination of carvedilol and enalapril. As a result, it was concluded that while enalapril alone may have attenuated further myocardial remodeling, carvedilolreversed the process, resulting in greater decrease in LVESVI and improvement in EF. This improvement in outcome was attributed to concurrent afterload reduction (*α*1-blockade) and blockade of adrenergic activation (combined *β*1-2) provided by carvedilol. The findings from the CARMEN study reinforce the importance of early comprehensive (combined *β*1-2, *α*1 blockade as offered by carvedilol) intervention for reversal of myocardial remodeling and neurohormonal imbalance in populations at risk for CHF.

 A recent study by Cardinale and colleagues [61] evaluated 201 consecutive patients with adult-onset malignancy and LVEF ≤45% due to anthracycline-associated cardiomyopathy. Enalapril and, when possible, carvedilol were initiated after detection of LVEF impairment. One hundred sixteen patients (58%) were partial or non-responders and 85 (42%) were complete responders, defined as recovery of LVEF. Responders were significantly more likely to have been treated with enalapril plus carvedilol when compared to partial or nonresponders (78% versus 53%, *P* < 0.01). Shorter duration to treatment initiation and low New York Heart Association (NYHA) functional class were found to be the only independent predictors of LVEF recovery. In fact, there was a four-fold decrease in likelihood of complete recovery of cardiac function for each doubling in time to treatment initiation, reinforcing the importance of cardiac screening and early initiation of comprehensive pharmacologic therapy in survivors with asymptomatic decline in LVEF.

 A retrospective review of 18 doxorubicin-exposed survivors of childhood cancer with stage B or C heart failure revealed that treatment with enalapril for a median of 10 years was associated with improvement in LV dimension, afterload, and systolic function in all patients [62]. However, the beneficial effects appeared to be transient. The 6 patients with symptomatic disease progressed to cardiac transplantation or death, reinforcing the previously reported poor outcomes when intervention is initiated after onset of clinically symptomatic disease. Of the 12 patients with asymptomatic disease, 3 developed heart failure or died. However, due to the relatively small number of asymptomatic individuals included in the study as well as the nonrandomized nature of the intervention, it was unclear what effect, if any, ACE-inhibition had in preventing progression to clinical CHF. At this time, the effectiveness of tertiary prevention in delaying onset of CHF in childhood cancer survivors with stage B disease remains unclear.

### 4.1. Future Directions

 The studies by Cardinale et al. [61] and Lipshultz et al. [62] highlight the importance of initiating pharmacologic intervention soon after detection of change in LVEF, prior to onset of symptomatic disease. The Children's Oncology Group long-term follow-up (LTFU) guidelines [36] recommend serial screening for LV dysfunction using echocardiograms at an interval of every 1–5 years depending on anthracycline dose, radiation therapy exposure, and age at cancer diagnosis (Table 2). These guidelines are risk-based, exposure-related clinical practice guidelines that rely on the epidemiological evidence of the association between therapeutic exposures and specific adverse outcomes, and are grounded in the collective experience of experts in the field of cancer survivorship. While prospective screening sets the stage for pharmacologic interventions prior to CHF, little is known regarding the utility and relevance of strategies advocated in the LTFU guidelines. With screening echocardiograms estimated to cost well over $1000 per test, [63, 64] and the intensity of screening not based on data from randomized clinical trials, it is imperative that we evaluate the cost-effectiveness of these screening practices, taking into consideration the potential effects on quantity as well as the quality of life affected by these screening practices.

## 5. Conclusions

 Cardiovascular complications remain a leading cause of morbidity and mortality in long-term survivors of childhood cancer. During the last two decades there has been a sustained effort to try and identify the clinical-and treatment-related risk factors for these outcomes. However, there continue to be large gaps in knowledge with regards to the strategies for prevention of therapy-related adverse events. These gaps can be filled only by approaching these problems in a systematic, comprehensive manner that not only helps identify those at highest risk of these adverse outcomes but also modifies the natural history of their disease. This approach requires multidisciplinary collaborations and access to large patient populations. Ongoing “cardiooncology” initiatives [65] help set the stage for such collaborations to minimize the burden of cardiovascular disease in survivors of pediatric and adult-onset malignancies.

## Figures and Tables

**Table 1:** Strategies for prevention of anthracycline-related congestive heart failure.

Type of prevention	Definition	Examples
Primary prevention	Preventing the initial development of a disease	(i) Limit lifetime anthracycline exposure (ii) Less cardiotoxic analogues (a) Epirubicin (b) Idarubicin (c) Mitoxantrone (iii) Alternative drug administration schedules (a) Bolus versus continuous infusion (iv) Cardioprotectants (a) Dexrazoxane

Secondary prevention	Prevention of disease before onset of signs and symptoms of illness	(i) Adoption of healthy lifestyle (ii) Aggressive management of modifiable risk factors (hypertension, diabetes) (iii) Pharmacologic intervention (a) ACE inhibitors (b) *β* blockers

Tertiary prevention	Reducing the impact of the disease	(i) Pharmacologic intervention (a) ACE inhibitors (b) *β* blockers

**Table 2:** Children's Oncology Group's recommended frequency of echocardiogram or MUGA scan for childhood cancer survivors.^∗^

Age at treatment^†^	Chest radiation	Anthracycline dose^††^	Recommended frequency
<1 year old	Yes	Any	Every year
	No	<200 mg/m^2^ ≥200 mg/m^2^	Every 2 years Every year

1–4 years old	Yes	Any	Every year
	No	<100 mg/m^2^ ≥100 to <300 mg/m^2^ ≥300 mg/m^2^	Every 5 years Every 2 years Every year

≥5 years old	Yes	<300 mg/m^2^ ≥300 mg/m^2^	Every 2 years Every year
	No	<200 mg/m^2^ ≥200 to <300 mg/m^2^ ≥300 mg/m^2^	Every 5 years Every 2 years Every year

Any age with decrease in serial function			Every year

^∗^From the Children's Oncology Group Long-Term FollowUp Guidelines for Survivors of Childhood, Adolescent, and Young Adult Cancers, Version 3.0, October 2008, used with permission.

^†^Age at time of first cardiotoxic therapy.

^††^Based on equivalent mg of doxorubicin/daunomycin.
